# Seed inoculation with antagonistic bacteria limits occurrence of *E. coli* O157:H7*gfp +* on baby spinach leaves

**DOI:** 10.1186/s12866-022-02550-w

**Published:** 2022-05-14

**Authors:** Maria E. Karlsson, Elisabeth Uhlig, Åsa Håkansson, Beatrix W. Alsanius

**Affiliations:** 1 Microbial Horticulture Division, Department of Biosystems and Technology, Swedish University of Agricultural Sciences, PO Box 190, 234 22 Lomma, SE Sweden; 2 Department of Food Technology, Engineering and Nutrition, Lund University, PO Box 124, SE- 22100 Lund, Sweden

**Keywords:** Bacterial antagonist, *E. coli* O157:H7, *Pseudomonas flavescens*, *Pseudomonas orientalis*, *Rhodococcus* sp., Spinach

## Abstract

**Backround:**

During the last decades, outbreaks of foodborne illnesses have increasingly been linked to fresh and/or minimally processed fruit and vegetables. Enterohemorrhagic *Escherichia coli* was the causal agent for major outbreaks in Europe with leafy green vegetables and sprouts. To improve food safety, microbial antagonism has received attention during recent years and could be one of the solution to prevent contamination of food borne pathogens on fresh produce. Here we investigate the antagonistic effect of three bacterial strains (*Pseudomonas orientalis*, *P. flavescens* and *Rhodococcus* sp.) isolated from spinach leaves against *E. coli* O157:H7*gfp +* under laboratory and greenhouse conditions.

**Results:**

Our results shows that significantly less culturable *E.coli* O157:H7*gfp +* were retrieved from the spinach canopy subjected to antagonist seed treatment than canopy inoculation. Seeds inoculated with *Rhodococcus* sp. significantly reduced growth of *E. coli* O157:H7*gfp +* compared with the other antagonists. The result from the *in vitro* study shows a significant reduction of growth of *E. coli* O157:H7*gfp+*, but only after 44 h when *E. coli* O157:H7*gfp +* was propagated in a mixture of spent media from all three antagonists.

**Conclusions:**

The antagonistic effect on phyllospheric *E.coli* O157:H7*gfp +* observed after seed inoculation with *Rhodococcus* sp. might be an indication of induced resistance mechanism in the crop. In addition, there was a small reduction of culturable *E.coli* O157:H7*gfp +* when propagated in spent media from all three antagonists. Nutritional conditions rather than metabolites formed by the three chosen organisms appear to be critical for controlling *E. coli* O157:H7*gfp+.*

## Backround

Consumption of fresh fruit and vegetables is associated with a healthy diet and lifestyle and has increased in popularity during the past decade. However, fresh produce is also recognised as a possible vehicle for transmission of food-borne human pathogens and the number of reported outbreaks is continually increasing [1–3]. Among these pathogens, *Escherichia coli* O157:H7 is the most frequently identified serotype [4]. To mitigate the occurrence and risks associated with shigatoxigenic *E. coli* (STEC) in horticultural value networks a hurdle approach could be used [5]. The hurdle technology is combining several methods that is not effective by its own but together can serve as a tool to reduce or even eliminate pathogens in food products [5]. Introduction into cultivation systems of microorganisms with antagonistic characteristics to food-borne pathogens is one such hurdle.

The phyllosphere as a habitat varies in structure and is constantly exposed to fluctuations in environmental factors such as temperature, humidity, nutrient availability and light [6–8]. Thus, its inhabitants need to possess a certain degree of plasticity to adapt to these changes [9]. The success of a microbe in its community is dependent how well it can compete with other community members [1]. For a human pathogen seeking to invade a habitat outside its normal niche, its fate is dependent on resource availability and the structure of the microbial community [10]. To successfully outcompete microbial invaders, such as shigatoxigenic *E. coli*, antagonists must: (i) share the same niche, (ii) have similar nutrition preferences to the undesired microbe, but a faster growth rate under ambient conditions, and (iii) fit into the existing microbial aggregate on the leaf surface. Formation of metabolites deleterious to the intruder under ambient conditions offers an additional mode to exclude microbial invaders.

This study investigated the potential for counteracting culturable *E. coli* O157:H7*gfp* + on spinach leaves by enrichment with three endemic phyllosphere bacteria. All three bacterial strains are known to display *in vitro* antagonistic properties against *E. coli* [11]. The objectives of this study was to investigate bacterial antagonism against *E. coli* O157:H7*gfp +* on spinach baby leaves using two different inoculation methods and also investigate *in vitro* and whether the metabolites of the antagonists could limit growth of *E. coli* O157:H7*gfp+*.

The hypotheses tested were that:

(i) Canopy spray, but not seed inoculation, limits the occurrence of culturable *E. coli* O157:H7*gfp +* on spinach leaves.
(ii) Metabolites from antagonists reduce the growth of *E. coli* O157:H7*gfp* + when propagated in broth with metabolites from the antagonists.

## Results

### Greenhouse experiment

**Fig. 1:**
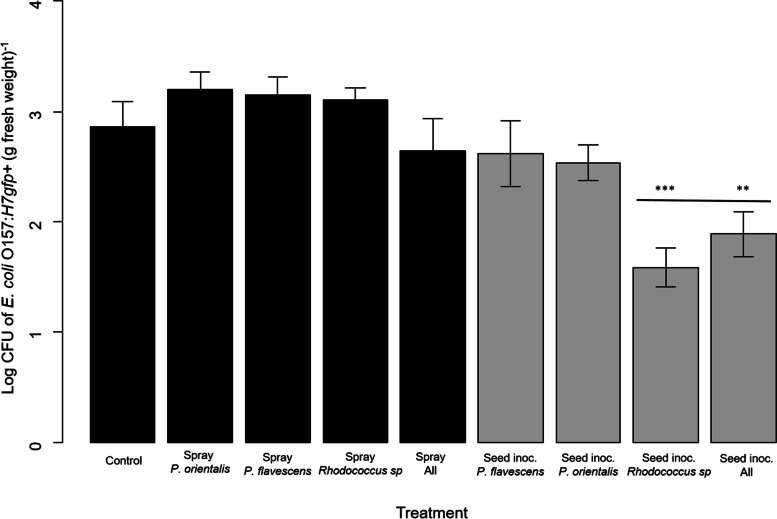
Log CFU of *Escherichia coli* O157:*H7gfp+* (g fresh weight)^−1^ isolated from spinach leaves at harvest and propagated on LB with ampicillin and arabinose (*N* = 12 per treatment). Error bars indicate standard error. Spinach was either seed- or spray-inoculated with three bacterial strains isolated from washed and bagged spinach with known antagonistic effects to *E. coli* O157:H7*gfp+.* Inoculation was performed as either a single-strain (*Pseudomonas orientalis*, *Pseudomonas flavescens*, *Rhodococcus* sp.) or multiple-strain (“inoc. all”) treatment. Black bars represent canopy inoculation by spraying. Grey bars represent seed inoculation. The results from the General linear model (GLM) are indicated with asterisk above bars. (*p *< 0.01*, *p* < 0.001**, *p* < 0.0001***)

Culturable *E. coli* O157:H7*gfp +* in the spinach canopy was found to be significantly reduced after seed inoculation than after canopy inoculation with the antagonists (Estimate (95%CI) -0.74 (-1.28 − 0.20). Seed inoculation with *Rhodococcus* sp. decreased phyllospheric *E. coli* O157:H7*gfp +* significantly, by log 1.45 CFU (g fresh weight)^−1^ (Estimate (95% CI) -1.28 (-1.87 – -0.69) compared with control plants (Fig. 1). Likewise, *E. coli* O157:H7*gfp+* (log CFU) were significantly lower when *E. coli* O157:H7*gfp +* was exposed to plants that were seed-inoculated with multiple bacterial strains (Estimate (95% CI) -1.07 (-0.78 – -0.35) (Fig. 1). No significant impact on culturable *E. coli* O157:H7*gfp +* was found after seed inoculation with either of the two *Pseudomonas* strains. Interestingly, despite two canopy sprays with the individual or multiple strain treatment, *E. coli* colonised spinach leaves to the same extent in the antagonist and the control treatment. There was a tendency for higher *E. coli* O157:H7*gfp +* counts in the single-strain antagonist treatments than in the treatment with a mixture of all three antagonists, but the differences were not significant.

### Impact of bacterial metabolites on growth of E. coli O157:H7gfp+

The initial nutrient concentration in the overnight culture medium was decisive for the growth of *E. coli* O157:H7*gfp +* in the spent media. The nutritional contribution from the metabolites seemed to promote rather than reduce the growth of *E. coli* O157:H7*gfp+*. This was also confirmed when calculating the generation time for *E. coli* O157:H7*gfp +* in all treatments except that with the spent media from *Rhodococcus*, where the generation time was shortened compared with the two control treatments (Table 1). Interestingly, the fastest generation time was seen in the multiple-strain treatment (Table 1). Treatment with spent media filtrate from *P. flavescens, P. orientalis* and the multiple-strain mixture reached the maximum number of *E. coli* O157:H7*gfp +* after only 22 h, whereas in the control this took 44 h (Table 1).

**Table 1:** Generation time, length of the log phase and time taken to reach maximum number of *Escherichia coli* O157:H7*gfp +* in the different treatments

	Generation time (h)	Length of log phase (h)	Time (h) to reach maximum number of *E. coli* O157:H7*gfp+*
Control 1 (no cell-free extract)	1 h 48 min	9	44
Control 2 (cell-free extract from *E. coli*)	1 h 29 min	9	26
*Pseudomonas flavescens*	1 h 14 min	4	22
*P. orientalis*	1 h	4	30
*Rhodococcus* sp.	1 h 50 min	4	22
Multiple-strain treatment	35 min	4	22

**Fig. 2:**
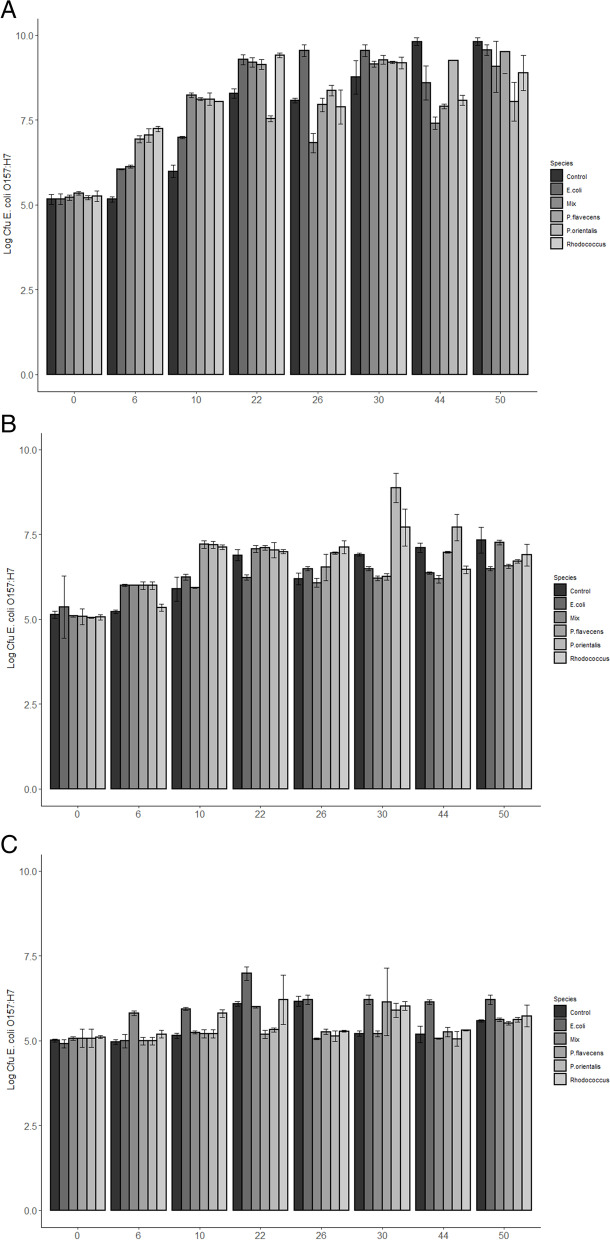
Viable counts (LogCFU with standard error bars, *N* = 6) of *Escherichia coli* O157:H7*gfp +* growth in (A) full-strength TSB and spent media filtrate; (B) 10% TSB and spent media filtrate and (C) 1% TSB and spent media filtrate. Control was *E. coli* O157:H7*gfp +* grown in LB media, E. coli was *E. coli* O157:H7*gfp +* grown in spent media filtrate of its own metabolites

When *E. coli* O157:H7*gfp +* was grown in full-strength TSB together with the multiple-strain spent media filtrate, a significant reduction in *E. coli* O157:H7*gfp +* numbers was observed after 44 h (Estimate (95% CI )-1.22 (-1.59 – -0.85), *p* < 0.001, Fig. 2 A). There was also a significant reduction of growth when *E. coli* O157:H7*gfp +* was propagated in the spent media filtrate of *Rhodococcus sp* (Estimate (95% CI)-1.73 (-2.10 – -1.36), *p* < 0.001, Fig. 2 A). When *E. coli* O157:H7*gfp +* was grown in low-concentration TSB (10%) with spent media filtrate, a significant decrease in growth was seen for the filtrate from *Rhodococcus* sp (Estimate (95% CI)-0.64 (-0.93 – -0.35), *p* < 0.001, Fig. 2B). and the multiple-strain treatment (Estimate (95% CI) -0.92 (-1.21 - -0.63), *p* < 0.001, Fig. 2B). With very low-concentration TSB (1%) and spent media filtrate, growth of *E. coli* O157:H7*gfp +* was reduced by approximately 1 log after 26 h in all treatments (Estimate (95% CI) *P. flavescens*: -0.90 (-1.07 – -0.73), *p* < 0.001, *P. orientalis*: -1.03 (-1.20 – -0.86), *p* < 0.001, *Rhodococcus sp*: -0.89 (-1.06 – -0.69), *p* < 0.001, Multiple-strain treatment: -1.11 (-1.28 – -0.94), *p* < 0.001). Hence, after 30 h the treatment promoted growth instead of inhibiting growth (Fig. 2 C).

## Discussion

Bacterial antagonism is not a new phenomenon. Lactobacilli have also been shown to have inhibitory effects on different pathovars of *E. coli* in vitro [12]. Both *Pseudomonas* strains and the *Rhodococcus* sp. strain used in the study originated from spinach leaves. Antagonistic potential of both genera is frequently mentioned in the literature with respect to various plant pathogens producing antibacterial substances [13–15]. In the present study, seed inoculation, but not canopy inoculation, with antagonistic bacterial strains under given conditions limited the presence of the inoculated model strain *E. coli* O157:H7*gfp+*, with *Rhodococcus* sp. decreasing numbers of the model strain by approximately log 1. This observation is not only of biological relevance but also of crop management relevance as it points to the possibility to mitigate this food pathogen independent of pre-harvest crop contamination events. Interestingly, *in vitro*-interactions between the three chosen bacterial strains and *E. coli* could not be repeated *in planta*. Given the fact that nutritional properties overruled the impact of potential secondary metabolites formed by the three chosen bacterial strains and the limited direct impact of canopy spray of the three chosen strains might indicate that metabolites formed by the three chosen strains might not be the essential for limiting *E. coli* O157:H7*gfp+*. Results retrieved from seed inoculation with *Rhodococcus* sp. indicate that induced resistance rather than niche exclusion might be mechanisms to further follow up on.

The 1 log reduction of *E. coli* O157:H7*gfp +* achieved by *Rhodococcus* sp. is encouraging and the differences observed are of biological relevance, but not of food safety relevance. *Rhodococcus* belongs to the phylum Actinobacteria, a Gram-positive bacteria commonly present in the environment. *Rhodococcus* also produces secondary metabolites that have an antibiotic effect on both Gram-positive and Gram-negative bacteria [16, 17]. However, the *Rhodococcus* strain used in the seed inoculation treatment does not substantially colonise the phyllosphere, so other mechanisms must affect the capacity of *E. coli* O157:H7*gfp +* to establish on spinach leaves, for example microbial community distortion towards a composition unfavourable to the invader or induced resistance plant mechanisms. In future studies, it would be highly interesting to follow the dynamics of *Rhodococcus* and to examine plant-related processes in the rhizosphere and canopy post seed inoculation. As the genus *Rhodococcus* is classified as a BSL2-organism, it is imperative to remove all possible doubts regarding its genetic make-up before it is tested against other serovars of shigatoxigenic *E. coli*.

The present study did not examine the capacity of the organisms to form secondary metabolites under chosen conditions (nutrient composition, temperature, length of pre-culture) or characterise such potential metabolites. To shed light on potential causes of decreased occurrence of the model strain, the impact of spent media extracts collected during the stationary phase on the model strain was examined. The spent media were collected from broth with different nutrient concentrations and a potential effect of metabolites was expected to emerge under nutrient-restricted conditions. However, production of secondary metabolites may not be produced in planktonic culture since it is strongly linked to biofilm formation mechanisms [18] and therefore it is doubtful that the antagonist in our study had enough time to produce biofilm and secondary metabolites only after 50 h. The results showed that nutritional conditions, rather than metabolite formation, were crucial for the growth of *E. coli* O157:H7*gfp+* (Fig. 2 A-C). Growth of *E. coli* O157:H7*gfp +* was influenced only to a very limited extent by secondary metabolites potentially formed by the three antagonists in the beginning of the stationary phase.

## Conclusions

Seed inoculation rather than canopy spray has the potential to mitigate the occurrence of culturable E. coli O157:H7gfp+, however, the choice of antagonist strain or multiple strain composition is essential. To explain the impact of seed inoculation with *Rhodococcus* sp. on *E. coli* O157:H7*gfp+*, induced resistance mechanisms should receive attention in future experiments. Nutritional conditions rather than metabolites formed by the three chosen organisms appear to be critical for controlling *E. coli* O157:H7*gfp+.*

## Materials and methods

A two-factor greenhouse experiment was set up, where factor 1 was treatmentwith antagonistic bacteria (*Pseudomonas orientalis*, *P. flavescens*, *Rhodococcus* sp.) and factor 2 was mode of treatment (seed or canopy inoculation). The model pathogen strain used was *E. coli* O157:H7 *gfp+* (verotoxin-1 negative, verotoxin-2 negative, *eae* gene positive) obtained from the Swedish Institute for Communicable Disease Control (Solna, Sweden), which was labelled with green fluorescent protein plasmid [19]. The three bacterial strains were isolated from spinach leaves in a previous study and identified with PCR and Sanger sequencing. The antagonistic activity against E.coli CCUG 29300T was determined with the perpendicular streak method [11].

### Inoculum preparation

The model pathogen *E. coli* O157:H7*gfp +* was propagated in LB agar supplemented with ampicillin and arabinose and incubated at 37℃ according to procedure described earlier in [20]. The three antagonists were propagated on TSA (Tryptic soy agar) plates and incubated overnight at 25 °C. A single colony was then transferred to 5 ml of TSB (Tryptic soy broth) and incubated overnight at 25 °C. The overnight cultures of the three antagonists and the pathogen were repeatedly washed with 0.85% NaCl and centrifuged at 3000 x g at 4 °C for 10 min (AvantiTM J-20 Centrifuge, Beckman Coulter Corporation, Brea CA, USA) and re-suspended in sterile NaCl (0.85% followed by 0.085%),Cell density was adjusted to OD 0.2 with NaCl (0.085%) which correspond to log 8.9 CFU mL^− 1^ for *P. orientalis* and *P. flavescens* and log 8.2 CFU mL^− 1^ for *Rhodococcus* ssp. Cell density of *E. coli* O157:H7*gfp +* was adjusted to OD 1 which correspond tolog 9.7 CFU mL^− 1^. For canopy inoculation, the final inoculum density of single- or multiple-strain suspensions was adjusted to log 7.7 with 0.085% NaCl. The volume of the spray inoculum was 150 ml. Multiple strain treatments consisted of equal numbers (CFU mL^− 1^) of the individual strains. For seed inoculation, 3 g of spinach seeds were imbibed overnight in 50 mL of the antagonist inoculum suspension at 25 °C.

### Greenhouse experiment

Spinach (*Spinacia oleraceae* cultivar SV157, seminis, UK ; 1830 seeds m^− 2^) was grown in peat-based growing medium (K-jord, Hasselfors Garden AB, Örebro, Sweden) for 30 days at a density of six replicates per treatment (set temperature: 20 °C (day), 16 °C (night); relative humidity: 70%; additional artificial light: 12 h, 118 µmol m^− 2^ s^− 1^). As control, spinach leaves were only inoculated with *E. coli* O157:H7*gfp +* without any treatment of the antagonists. The experiment were performed twice.

Canopy inoculation of the antagonistic strains occurred on day 23 after sowing and on day 28 after sowing. Canopy spraying with *E. coli* O157:H7*gfp +* was performed one day before harvest of the spinach leaves. Spinach leaves were harvested aseptically and leaf-associated microbes were extracted as previously described by [21]. Briefly, leaves were transferred to a sterile plastic bag with filter (Separator 400, Grade products Ltd., Coalville, UK) and 50 ml of TRIS-buffer (0.01 M Trishydroxymethyl, pH 8,) was added. The bag was placed in a smasher lab blender (Biomérieux Industry, Durnham NC, USA) for 30 s normal mode. The extraction suspension was 10-fold serially diluted in NaCl solution (0.85%). For viable counts, *E. coli* O157:H7*gfp +* was cultured on LB agar supplemented with ampicillin (100 µg mL^− 1^) and L-arabinose (0.2 g mL^− 1^) at 37 ℃ and fluorescent colonies were enumerated after 18 h under UV light. The *Pseudomonas* and *Rhodococcus* isolates were re-isolated on King Agar B (Merck) and full-strength TSA, respectively, supplemented with rifampicin (100 µg mL^− 1^) at 25℃ for 24 h.

### *Impact of metabolites of antagonistic bacterial strains on growth of E. coli* O157:H7*gfp+*.

The potential impact of metabolites of *P. flavescens, P. orientalis* and *Rhodococcus* sp. on *E. coli* O157:H7*gfp +* was tested in in vitro assays in a two-factor experiment, using the supernatants from overnight cultures of the three antagonists (factor 1) and three concentrations of the propagation broth (0.01x, 0.1x, 1x; factor 2) and with six replicates. The antagonists and *E. coli* O157:*H7gfp +* was propagated in three concentrations of TSB overnight. The supernatant was membrane-filtered (syringe filter 0.2 μm, Thermo Fisher) after centrifugation for 10 min at 3200 x g to remove remaining cells that were not collected by centrifugation. This treatment will not lyse the cells but removes them from the supernatant. This filtrate is referred to as spent media [22] Cells from an overnight culture of *E. coli* O157:H7*gfp +* were washed in 0.85% NaCl through repeated twice centrifugation (3200 xg for 10 min), finally suspended in LB and cell density was adjusted to 5.3 log CFU mL^− 1^ (OD_620_). Aliquots of 5 mL of membrane-filtered antagonist suspension were inoculated with 100 µL of *E. coli* O157:H7*gfp+*. Spent media from an overnight culture of *E. coli* O157:H7*gfp +* was used as a first control and *E. coli* O157:H7*gfp +* in LB media without any spent media filtrate as a second control. All tubes were incubated at 25 °C. Culturable *E. coli* O157:H7*gfp +* was enumerated after 6, 10, 22, 26, 30, 44 and 50 h. Plates were incubated at 25 °C and analysed after 24 h.

### Statistical analysis

Generalised linear model was used to investigate the effect of each antagonist and the mode of application on the survival of *E. coli* O157:H7*gfp +* on spinach leaves. Log-transformed number of culturable *E. coli* O157:H7*gfp+* (log CFU + 1) was used as the response variable and treatment and inoculation method as co-variates. The analysis were performed in R 3.5.3 (R Core Team, 2019). In the in vitro study, *E. coli* O157:H7*gfp +* propagated in its own metabolites was used as the response variable and treatment with the metabolites from the antagonists as co-variate.

## Data Availability

The data that support the findings of this study are available from the corresponding author upon reasonable request.

## References

[1] Brandl MT (2006). Fitness of human enteric pathogens on plants and implications for food safety. Annual Rev Phytopathology.

[2] Matthews KR, Sapers GM, Gerba CP (2014). The produce contamination problem.

[3] Alsanius BW (2014). Mikrobiologiska faror i grönsakskedjan under primärproduktion. Landskap Trädgård Jordbruk, Rapportserie. Vol.

[4] EFSA. Panel on Biological Hazards (BIOHAZ). Scientific opinion on the risk posed by pathogens in food of non-animal origin. Part 1 (outbreak data analysis and risk ranking of food/pathogen combinations. EFSA journal, 2013;11(1):3025 DOI: 10.2903/j.efsa.2013.3025 10.2903/j.efsa.2013.3025 PMC13159936 42124987

[5] Mogren L, Windstam S, Boqvist S, Vågsholm I, Söderqvist K, Rosberg AK, Lindén J, Mulaosmanovic E, Karlsson ME, Uhlig E, Håkansson Å, Alsanius BW. The hurdle approach - A holistic concept for controlling food safety risks associated with pathogenic bacterial contamination of leafy green vegetables. A review. Frontiers in microbiology, 2018; 9, 1965. DOI:10.3389/fmicb.2018.01965 10.3389/fmicb.2018.01965 PMC6117429 30197634

[6] Lindow SE, Brandl MT (2003). Microbiology of the phyllosphere. Appl Environ Microbiol.

[7] Vorholt JA. Microbial life in the phyllosphere. Nat Rev Microbiol. 2012; Dec;10(12):828 – 40. doi: 10.1038/nrmicro2910. PMID: 23154261. 10.1038/nrmicro2910 23154261

[8] Leveau JHJ (2019). A brief from the leaf: latest research to inform our understanding of the phyllosphere microbiome,Current Opinion. in Microbiology.

[9] Leveau JHJ (2006). Microbial communities in the phyllosphere. Biology of plant cuticle ed. Riederer M. and Müller C.

[10] Hawkes CV, Connor EW (2017). Translating phytobiomes from theory to practice: Ecological and evolutionary considerations. Phytobiomes Journal.

[11] Uhlig E, Kjellström A, Nurminen N, Olsson C, Canaviri-Paz P, Mogren L, Alsanius BW, Molin G, Håkansson Å (2021). Use of bacterial strains antagonistic to *Escherichia coli* for biocontrol of spinach: A field trial. Innovative Food Science and Emerging Technologies.

[12] Karimi S, Rashidian E, Birjandi M, Mahmoodnia L (2018). Antagonistic effect of isolated probiotic bacteria from natural sources against intestinal Escherichia coli pathotypes. Electronic physician.

[13] Hong CE, Park JM (2016). Endophytic bacteria as biocontrol agents against plant pathogens: current state-of-the-art. Plant Biotechnology Reports.

[14] Duraira K, Velmurugan P, Park J, Chang W, Park Y, Senthilkumar P, Choi K, Lee J, Oh B. Potential for plant biocontrol activity of isolated Pseudomonas aeruginosa and Bacillus stratosphericus strains against bacterial pathogens acting through both induced plant resistance and direct antagonism, FEMS Microbiology Letters, 2018; 364 (23), 10.1093/femsle/fnx225 10.1093/femsle/fnx225 29069329

[15] Zengerer V, Schmid M, Bieri M, Müller DC, Remus-Emsermann MNP, Ahrens CH, Pelludat C (2018). Pseudomonas orientalis F9: A potent antagonist against phytopathogens with phytotoxic effect in the apple flower. Front Microbiol.

[16] Kitagawa W, Tamura T (2008). A quinoline antibiotic from Rhodococcus erythropolis JCM 6824. J Antibiot..

[17] Kitagawa W, Tamura T (2008). Three types of antibiotics produced from Rhodococcus erythropolis strains. Microbes Environ..

[18] Rieusset L, Rey M, Muller D, Vacheron J, Gerin F, Dubost A, Comte G, Prigent-Combaret C (2020). Secondary metabolites from plant-associated Pseudomonas are overproduced in biofilm. Microbial Biotechnology..

[19] El-Mogy MM, Alsanius BW (2012). Cassia oil for controlling plant and human pathogens on fresh strawberries. Food Control.

[20] Alam M, Ahlström C, Burleigh S, Olsson C, Ahrné S, El-Mogy M, Molin G, Jensen P, Hultberg M, Alsanius BW (2014). Prevalence of Escherichia coli O157:H7 on spinach and rocket as affected by inoculum and time to harvest. Scientia Horticulturæ.

[21] Darlison J, Mogren L, Rosberg AK, Grudén M, Minet A, Liné C, Mieli M, Begtsson T, Håkansson Å, Uhlig E, Becher PG, Karlsson ME, Alsanius BW (2019). Leaf mineral content govern microbial community structure in the phyllospher of spinach (*Spinacia oleracea)* and rocket (*Diplotaxis tenuifolia*). Science of the total environment.

[22] Lopez-Valesco G, Tydings HA, Boyer R, Falkinham JO, Ponder MA (2012). Characterization of interactions between *Escherichia coli* O157:H7 with epiphytic bacteria *in vitro* and on spinach leaf surfaces. International Journal of food Microbiology.

